# Transient ischaemic attack and ischaemic stroke: constructing episodes of care using hospital claims data

**DOI:** 10.1186/1756-0500-6-128

**Published:** 2013-04-02

**Authors:** Janet K Sluggett, Gillian E Caughey, Michael B Ward, Elizabeth E Roughead, Andrew L Gilbert

**Affiliations:** 1 Quality Use of Medicines and Pharmacy Research Centre, Sansom Institute for Health Research, University of South Australia, GPO Box 2471, Adelaide, SA, 5001, Australia; 2 School of Pharmacy and Medical Sciences, University of South Australia, GPO Box 2471, Adelaide, SA, 5001, Australia

**Keywords:** Ischaemic stroke, Transient ischaemic attack, Hospitalisation, Administrative health claims data

## Abstract

**Background:**

Stroke patients may have multiple hospital separations relating to the same stroke. Understanding the pattern of hospitalisations for these patients enables first and recurrent events to be distinguished to better understand care. The aim of this study was to investigate reasons for hospital separations after transient ischaemic attack (TIA) or ischaemic stroke and construct episode of care criteria.

**Methods:**

A retrospective observational study was conducted using the Australian Government Department of Veterans’ Affairs administrative claims database. All patients hospitalised for TIA or ischaemic stroke in 2008–2009 were included. Reasons for hospital separations in the 60 days after TIA or ischaemic stroke were classified by a clinical panel as ‘probably’, ‘possibly’ or ‘unlikely’ to be related to the index separation. Based on panel assessment and time between separations, episode of care criteria for TIA and ischaemic stroke were constructed.

**Results:**

Of the 4520 veterans alive after the index separation, 32% of TIA patients (n=782) and 63% of ischaemic stroke patients (n=1323) had another separation within 60 days. The clinical panel reviewed 460 unique reasons for readmission. Of the 3263 separations, 55% and 85% were classified as related to the index TIA and ischaemic stroke separation, respectively.

**Conclusions:**

Patients hospitalised for ischaemic stroke are likely to have multiple hospital separations for treatment of the same event. Multiple separations for treatment of TIA were less frequent. Consideration of these related separations is recommended when assessing health service utilisation from claims databases.

## Background

Patients hospitalised with ischaemic stroke are known to have complicated care transitions [1]. Medical complications, hospital transfers and extended periods of post-acute treatment, such as rehabilitation, are common [2,3]. As a result, these patients may have multiple hospital separations recorded for treatment of the same stroke [4]. Patients hospitalised with ischaemic stroke or transient ischaemic attack (TIA) are also at risk of recurrent events [5]. Recurrent TIAs can occur within a short time, an ischaemic stroke can occur soon after TIA [5], and both types of ischaemic events can be followed by haemorrhagic complications [3].

Given these complexities, consideration of related separations and distinguishing between same and recurrent events when using administrative health claims data is particularly important for these patient groups. Similar to other countries, Australian hospital claims datasets contain records of hospital separations; not entire hospitalisations or completed events. Hospital separations are recorded at the time of patient discharge, death, hospital transfer or when there is a change in the type of care [6]. Little is known about the pattern of separations after admission to hospital for TIA or ischaemic stroke, and there is no consensus on appropriate decision rules to link related separations and define same and recurrent events [7]. Studies accounting for multiple separations have used decision rules to link claims for patients readmitted on the same day [8,9] or transferred between hospitals [10]. Intervals of up to year after a first TIA or ischaemic stroke have been used before a subsequent event is defined as recurrent [7,11-13].

Understanding the pattern of separations for these patients enables first and recurrent events to be distinguished to better understand care. The aim of this study was to investigate reasons for hospital separations after TIA or ischaemic stroke and construct episode of care criteria for use in administrative health claims datasets.

## Methods

A retrospective observational study was conducted using data from the Australian Government Department of Veterans’ Affairs (DVA) administrative health claims database. This database contains details of all hospital separations, medical and allied health services and prescription medicines subsidised by DVA, for a treatment population of 258,000 veterans and dependents. Over 70% of the population are aged 70 years or older, 58% are male and 9.8% live in residential aged care [14]. Hospitalisations are coded according to the World Health Organisation (WHO) International classification of diseases, 10^th^ revision (ICD-10), Australian modification [15]. Date of death is determined from death notices, family notifications and the Australian Government Births, Deaths and Marriages registries.

The study included all persons hospitalised with a primary diagnosis of TIA (ICD-10AM codes G45.0, G45.1, G45.2, G45.8, or G45.9) or ischaemic stroke (ICD-10AM code I63) between 1^st^ January 2008 and 31^st^ December 2009. Subjects were eligible for all health services subsidised by DVA. Where more than one separation for TIA or ischaemic stroke was recorded for a subject during the study period, analysis was limited to the first separation during the study period (this separation is referred to as the index separation).

For subjects alive after the index separation, all hospital separations within 60 days were extracted. This time period was chosen to enable inclusion of all separations associated with acute stroke treatment and rehabilitation, based on previous reports of average length of stay for stroke rehabilitation [4,16]. A list of unique reasons for hospital separations after TIA or ischaemic stroke was prepared from the primary diagnosis codes. The list included both the ICD-10AM code and the detailed description of the code. Similar ICD codes were listed together where appropriate; for instance, any primary diagnosis from the ICD-10AM classifications E10 – E14 was classed under ‘diabetes mellitus’. Where the diagnosis was for transient ischaemic attack or stroke, this was listed up to three times; each listing indicating the time elapsed since the index separation (0 – 1 day, 2 – 7 days, or 8 to 60 days).

A panel of three practicing clinicians (neurologist, clinical pharmacologist and clinical pharmacist) independently assessed the reasons for separations. Using clinical judgement, panel members were asked to classify separations as “probably” related where the diagnosis was for acute stroke care or for a known complication with a direct pathological link to acute TIA or ischaemic stroke; “possibly” related when the diagnosis was a known stroke-related complication which may be diagnosed independently of acute TIA or ischaemic stroke; or “unlikely” when there was no pathological link between the diagnosis and acute TIA or ischaemic stroke. Kendall’s coefficient of concordance was calculated to measure agreement between members of the clinical panel before resolving disagreement by discussion [17,18]. The final classification for each diagnosis code was applied to the dataset for analysis.

Descriptive statistics were used to report the characteristics of the study population and the reasons for hospital separations in the 60 day follow-up period. All analyses were performed using SAS version 9.2 (SAS Institute Inc., Cary, NC, USA). Ethics approval was gained from the University of South Australia and DVA Human Research Ethics Committees.

## Results

Characteristics of the 4882 patients included in this study are described in Table 1. Of the 4520 patients alive after the index separation, 2105 (47%) had another hospital separation within 60 days. The number of separations for these subjects ranged from one to 27, with a total of 3263 separations included for analysis.

**Table 1:** Subject characteristics

	**TIA**	**Ischaemic stroke**
Number of subjects	2443	2439
Age at admission (years)		
Mean (± SD)	85.0 (± 6.4)	85.3 (± 6.2)
Median (IQR)	85.7 (82.8–88.6)	86.1 (83.2–88.7)
Male gender (%)	1263 (51.7%)	1224 (50.2%)
Died during index separation (%)	18 (0.7%)	344 (14.1%)
Died within 60 days of index separation (%)	106 (4.3%)	584 (23.9%)
Number of subjects with another separation within 60 days (% discharged alive)	782 (32.2%)	1323 (63.1%)
Time between separations (days), median (IQR)	2 (0 – 15)	0 (0 – 0)

Of the 3263 separations recorded, 559 (17%) were for TIA or any stroke type. Of these, 410 (73%) were readmitted within one day of the index separation, 501 (90%) were readmitted within 14 days, and 532 (95%) were readmitted within 28 days.

Of the 3263 hospital separations, a total of 460 unique diagnoses were identified for review by the clinical panel. Of these, the panel classified 63 (14%) as ‘probably’, 83 (18%) as ‘possibly’ and 314 (68%) as ‘unlikely’ to be related to the index separation. The clinical panel classified separations for TIA or stroke readmitted within 0 – 1 day after the index separation as ‘probably’ related. All other separations for TIA or stroke were classified as ‘possibly’ related. There was moderate agreement amongst panel members when assessing the relationship between the index separation and the reasons for separations in the follow-up period (Kendall’s coefficient of concordance=0.49; p-value <0.0001). A complete list of the diagnoses classified by the clinical panel as ‘probably’, ‘possibly’ or ‘unlikely’ to be related to the index separation are provided in additional files (see Additional files 1, 2, and 3).

The majority of separations after ischaemic stroke were classified as stroke-related (75% ‘probable’, 10% ‘possible’). For patients hospitalised with a TIA, approximately half of all separations were classified as related (31% ‘probable’, 23% ‘possible’). The most frequently occurring diagnoses classified as ‘probable’ or ‘possible’ by the clinical panel are listed in Table 2.

**Table 2:** Most frequent “probable” or “possible” reasons for separations after TIA or ischaemic stroke

	**ICD-10AM codes**	**TIA**	**Ischaemic stroke**
Total number of separations within 60 days		1274	1989
*Reasons for separations classified as “probably” related (% of all separations)*			
Rehabilitation	Z50	82 (14.3%)	1000 (50.3%)
Stroke, within 0 – 1 day	I60–I64	31 (2.4%)	288 (14.5%)
TIA, within 0 – 1 day	G45	84 (6.6%)	7 (0.4%)
Awaiting bed elsewhere	Z75.1	41 (3.1%)	112 (5.6%)
*Reasons for separations classified as “possibly” related (% of all separations)*			
Stroke, within 2 – 60 days	I60–I64	33 (2.6%)	46 (2.3%)
TIA, within 2 – 60 days	G45	57 (4.5%)	13 (0.7%)
Cardiac arrhythmias	I44, I46–I49	26 (2.0%)	22 (1.1%)
Acute respiratory infection or pneumonia	J06, J12–J18, J22	35 (2.7%)	18 (0.9%)

**Figure 1:**
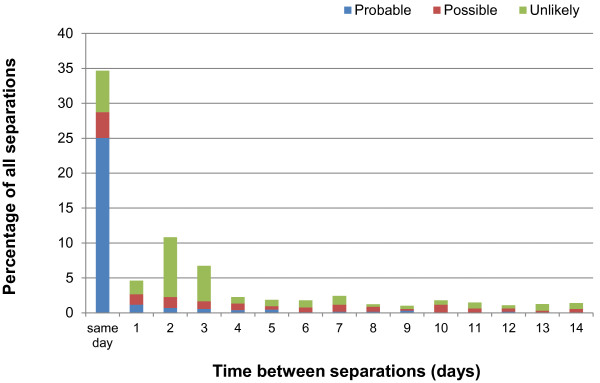
**Time between separations after transient ischaemic attack, according to clinical panel rating.** The first 14 days are shown.

**Figure 2:**
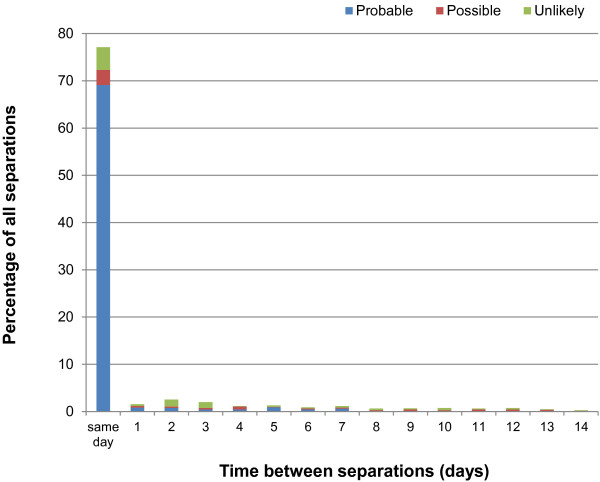
**Time between separations after ischaemic stroke, according to clinical panel rating.** The first 14 days are shown.

Examination of the time between separations showed approximately 35% of all hospital separations after TIA (Figure 1) and 75% of all separations after ischaemic stroke (Figure 2) were admitted within 24 hours of discharge.

## Discussion

This study confirms that patients hospitalised for ischaemic stroke are likely to have multiple hospital separations recorded for treatment of the same event. Two-thirds of ischaemic stroke patients had another hospital separation within 60 days, and 85% of these separations were stroke-related. Although TIA patients may also have multiple separations for treatment of the same event, this occurred less frequently than for ischaemic stroke patients. Our analysis indicates linkage of separations is appropriate when ischaemic stroke patients are discharged and readmitted within 24 hours, which is in agreement with a decision rule previously applied to Australian claims data [9].

In this study, a clinical panel was asked to independently assess reasons for hospital separations during the follow-up period. Expert opinion has guided stroke episode of care definitions in previous claims analysis [19] but the methodology and findings have not been described in detail. For research conducted in other clinical situations, grouping software [20], clinical panels [21], and other data based approaches [22-24] have been used to construct episodes of care and determine related outcome events.

Recent studies have shown failure to account for multiple separations may result in an underestimation of length of stay, hospitalisation costs, in-hospital mortality rates and readmission rates [23,24]. In a cohort of Australian veterans with hip fracture, examination of outcomes before and after linkage of separations showed increases in the average length of stay (from 11.1 days to 30.8 days), hospitalisation costs (from $AUD 13095 to $AUD 26023) and in-hospital mortality rate (from 6.5% to 11.1%) when separations were linked [24]. Sixty percent of veterans with hip fracture had multiple separations recorded for care of the same event [24]. In another study, failure to account for multiple separations for those admitted to intensive care overestimated the number of episodes of care by up to 10%, and underestimated the average length of stay by up to 30% [23]. The findings of the present study can be used to link related hospital separations in administrative health claims data to examine acute care and related outcomes for patients with TIA or ischaemic stroke. The impact of linking stroke-related separations on length of stay and hospitalisation costs may be significant, given the high incidence of stroke in the older population (almost 4,000 strokes per 100,000 Australians aged ≥85 years, per year [25]). Following linkage of related separations, final discharge dates can be used to determine when to commence review of secondary care for TIA and ischaemic stroke patients, such as examination of claims for secondary stroke prevention medicines and visits to medical practitioners.

In this study, the large sample size enabled the most common reasons for separations after TIA or ischaemic stroke to be identified. Although it can be argued some ICD codes provide limited information about the primary reason for admission, the diagnoses assessed as ‘probable’ and ‘possible’ were consistent with known stroke complications [3] and recommended post-stroke treatment. Whilst potential misclassification is possible, we asked three clinicians of different backgrounds to assess the reasons for admission, and checked concordance. Although we did not validate findings against medical records, the relationship between the index and next separation may not be well described in case notes, meaning clinical panel assessment would still be necessary.

One of the potential limitations in using claims data to examine TIA and ischaemic stroke management is incorrect selection of cases due to coding inaccuracies. Although ICD-9 codes for stroke have been extensively validated, assessment of ICD-10 coding for TIA and ischaemic stroke is limited [26]. A study comparing TIA and stroke coding after switching from the ICD-9 to ICD-10 system found the positive predictive value (PPV) of TIA coding using the ICD-10 system was 97% (95% CI: 88 – 99) versus 70% (95% CI: 56–82) for ICD-9 [27]. For both systems, ischaemic stroke was accurately coded 85% of the time (95% CI: 76 – 92 for ICD-10; 78 – 90 for ICD-9) [27]. In Australia, the reliability of ICD-10 coding is high; coders receive standardised training and coding quality is regularly assessed [9,28]. To improve accurate selection of cases we used similar inclusion criteria to other studies using ICD-10 data [27]; only primary diagnoses were used to select subjects, and we excluded those with other stroke types (I60-I62, I64-I69), amaurosis fugax (G45.3) and transient global amnesia (G45.4).

Results obtained from this study are likely to be representative of the older Australian population. Almost one quarter of Australians aged over 85 years are entitled to access DVA-funded services, and age-specific comparisons show veterans without service-related disabilities have similar use of health services and prescription medicines to the rest of the Australian population [14,29]. Given this, we expect our findings could also be applied to stroke-related hospitalisation records for older patients in other administrative health claims datasets.

## Conclusions

Analysis of Australian hospitalisation data indicates TIA and ischaemic stroke are associated with a cluster of related separations which are appropriate for linkage. Study findings can be used to link separations in health administrative datasets to form completed episodes of care, to better explore the appropriateness of medicines and health service utilisation after TIA or ischaemic stroke.

## Abbreviations

DVA: Department of Veterans’ Affairs; ICD-9: International classification of diseases, 9^th^ revision; ICD-10: International classification of diseases, 10^th^ revision; PPV: Positive predictive value; TIA: Transient ischaemic attack; WHO: World Health Organisation.

## Competing interests

The authors declare that they have no competing interests.

## Authors’ contributions

All authors participated in the design and interpretation of this study. JS performed the data analysis and drafted the manuscript. All authors contributed to the revision of the draft and have approved the final manuscript.

## Supplementary Material

Additional file 1 Reasons for separations after TIA or ischaemic stroke classified by the clinical panel as “probably” related. Click here for file

Additional file 2 Reasons for separations after TIA or ischaemic stroke classified by the clinical panel as “possibly” related. Click here for file

Additional file 3 Reasons for separations after TIA or ischaemic stroke classified by the clinical panel as “unlikely” to be related. Click here for file
