# Supplementary material for: Transient ischaemic attack and ischaemic stroke: constructing episodes of care using hospital claims data
Source: BMC Res Notes. 2013 Apr 2;6:128. doi: 10.1186/1756-0500-6-128 (PMC3620927; doi:10.1186/1756-0500-6-128)
Supplement: Additional file 1 — Reasons for separations after TIA or ischaemic stroke classified by the clinical panel as “probably” related. [file 1756-0500-6-128-S1.docx]

**Additional file 1.**

Reasons for separations after TIA or ischaemic stroke classified by the clinical panel as “probably” related.

| **Primary diagnosis** | **ICD10-AM codes** |
| --- | --- |
| Acute post haemorrhagic anaemia | D62 |
| Epilepsy or status epilepticus | G40–G41 |
| TIA, readmitted 0 – 1 day after discharge | G45 |
| Hemiplegia | G81 |
| Monoplegia | G83.3 |
| Ptosis of eyelid | H02.4 |
| Visual disturbances | H53 |
| Any stroke type, readmitted 0 – 1 day after discharge | I60–I64 |
| Occlusion and stenosis of pre-cerebral or cerebral arteries, readmitted 0 – 1 day after discharge | I65–I66 |
| Other cerebrovascular diseases | I67 |
| Sequelae of cerebrovascular disease | I69 |
| Pneumonitis due to food and vomit | J69.0 |
| Neuromuscular bladder dysfunction | N31 |
| Atrial septal defect | Q21.1 |
| Arteriovenous malformation of cerebral vessels | Q28.2 |
| Dysphagia | R13 |
| Neurologic neglect | R29.5 |
| Speech and voice disturbances | R47, R49 |
| Convulsions | R56.8 |
| Multiple intracerebral and cerebellar haematomas | S06.23 |
| Focal cerebellar haematoma | S06.33 |
| Care involving use of rehabilitation procedures | Z50.1, Z50.7–Z50.9 |
| Convalescence | Z54.0, Z54.7–Z4.9 |
| Problems related to care-provider dependency | Z74 |
| Problems related to medical facilities and other health care | Z75 |
