# Supplementary material for: Transient ischaemic attack and ischaemic stroke: constructing episodes of care using hospital claims data
Source: BMC Res Notes. 2013 Apr 2;6:128. doi: 10.1186/1756-0500-6-128 (PMC3620927; doi:10.1186/1756-0500-6-128)
Supplement: Additional file 2 — Reasons for separations after TIA or ischaemic stroke classified by the clinical panel as “possibly” related. [file 1756-0500-6-128-S2.docx]

**Additional file 2.**

Reasons for separations after TIA or ischaemic stroke classified by the clinical panel as “possibly” related.

| **Primary diagnosis** | **ICD10-AM codes** |
| --- | --- |
| Sepsis | A40–A41 |
| Unspecified malnutrition | E46 |
| Pure hypercholesterolaemia | E78.0 |
| Vascular dementia | F01 |
| Delirium | F05 |
| Personality and behavioural disorders due to brain disease, damage and dysfunction | F07 |
| Depressive episode | F32 |
| TIA, readmitted 2 – 60 days after discharge | G45 |
| Other mononeuropathies | G58 |
| Drug induced myopathy | G72.0 |
| Multi-system degeneration | G90.3 |
| Anoxic brain damage | G93.1 |
| Other post-procedural disorders of nervous system | G97.8 |
| Essential hypertension | I10 |
| Pulmonary embolism | I26 |
| Cardiac arrhythmias and conduction disorders | I44, I46–I49 |
| Stroke, readmitted 2 – 60 days after discharge | I60–I64 |
| Occlusion and stenosis of pre-cerebral or cerebral arteries, readmitted 2 – 60 days after discharge | I65–I66 |
| Arterial embolism and thrombosis | I74 |
| Acute upper respiratory infection, unspecified | J06.9 |
| Pneumonia, unspecified | J18.9 |
| Unspecified acute lower respiratory infection | J22 |
| Acute or unspecified respiratory failure | J96.0, J96.9 |
| Gastric or duodenal ulcer, with haemorrhage | K25, K26 |
| Haematemesis, malaena or haemorrhage | K92.0-K92.2 |
| Other bladder disorder | N32.8 |
| Urinary tract infection | N39.0 |
| Abnormalities of heart beat | R00 |
| Cardiac murmurs | R01 |
| Epistaxis | R04.0 |
| Other abnormalities of gait and mobility | R26.8 |
| Tendency to fall | R29.6, R29.81 |
| Coma | R40.2 |
| Dizziness and giddiness | R42 |
| Headache | R51 |
| Syncope and collapse | R55 |
| Haemorrhage, not elsewhere classified | R58 |
| Traumatic subdural or subarachnoid haemorrhage | S06.5, S06.6 |
| Other intracranial injuries | S06.8 |
| Haemorrhage and haematoma complicating a procedure | T81.0 |
| Shock during or resulting from a procedure | T81.1 |
| Adjustment and management of cardiac pacemaker | Z45.0 |
| Fitting and adjusting of urinary device | Z46.6 |
