# Supplementary material for: Transient ischaemic attack and ischaemic stroke: constructing episodes of care using hospital claims data
Source: BMC Res Notes. 2013 Apr 2;6:128. doi: 10.1186/1756-0500-6-128 (PMC3620927; doi:10.1186/1756-0500-6-128)
Supplement: Additional file 3 — Reasons for separations after TIA or ischaemic stroke classified by the clinical panel as “unlikely” to be related. [file 1756-0500-6-128-S3.docx]

**Additional file 3.**

Reasons for separations after TIA or ischaemic stroke classified by the clinical panel as “unlikely” to be related.

| **Primary diagnosis** | **ICD10-AM codes** |
| --- | --- |
| Enterocolitis due to Clostridium difficile | A04.7 |
| Viral and other specified intestinal infections | A08 |
| Gastroenteritis and colitis of unspecified origin | A09.9 |
| Tinea pedis | B35.3 |
| Candidiasis | B37 |
| Scabies | B86 |
| Streptococcus and staphylococcus as the cause of diseases classified to other chapters | B95 |
| Other specified bacterial agents as the cause of diseases classified to other chapters | B96 |
| Neoplasms | C00–D48 |
| Iron deficiency anaemia | D50 |
| Anaemia, unspecified | D64.9 |
| Hereditary deficiency of other clotting factors | D68.2 |
| Thrombocytopenia, unspecified | D69.6 |
| Secondary polycythaemia | D75.1 |
| Diabetes mellitus | E10–E14 |
| Syndrome of inappropriate secretion of antidiuretic hormone | E22.2 |
| Diabetes insipidus | E23.2 |
| Deficiency of other specified B group vitamins | E53.8 |
| Iron deficiency | E61.1 |
| Disorders of calcium metabolism | E83.5 |
| Volume depletion | E86 |
| Other disorders of fluid, electrolyte and acid-base balance | E87 |
| Dementia in Alzheimer disease, unspecified | F00.9 |
| Unspecified dementia | F03 |
| Mental and behavioural disorders due to use of alcohol | F10 |
| Delusional disorder | F22.0 |
| Mixed anxiety and depressive disorder | F41.2 |
| Anxiety disorder, unspecified | F41.9 |
| Post-traumatic stress disorder | F43.1 |
| Parkinson disease | G20 |
| Alzheimer’s disease, unspecified | G30.9 |
| Other degenerative diseases of nervous system, not elsewhere classified | G31 |
| Migraine | G43 |
| Sleep disorders | G47 |
| Bell palsy | G51.0 |
| Carpal tunnel syndrome | G56.0 |
| Polyneuropathy, unspecified | G62.9 |
| Normal-pressure hydrocephalus | G91.2 |
| Other specified disorders of brain | G93.9 |
| Entropion and trichiasis of eyelid | H02.0 |
| Ectropion of eyelid | H02.1 |
| Stenosis and insufficiency of lacrimal passages | H04.5 |
| Cataract | H25–H26 |
| Retinal detachment with retinal break | H33.0 |
| Degeneration of macula and posterior pole | H35.3 |
| Disorders of vestibular function | H81 |
| Labyrinthitis | H83.0 |
| Ischaemic heart diseases | I20–I25 |
| Other pulmonary heart diseases | I27 |
| Acute and subacute infective endocarditis | I33.0 |
| Aortic (valve) stenosis | I35.0 |
| Dilated cardiomyopathy | I42.0 |
| Heart failure | I50 |
| Cardiomegaly | I51.7 |
| Atherosclerosis | I70 |
| Thoracic aortic aneurysm, without mention of rupture | I71.2 |
| Aneurysm and dissection of iliac artery | I72.3 |
| Embolism and thrombosis of abdominal aorta | I74.0 |
| Other specified disorders of arteries and arterioles | I77.8 |
| Haemorrhoids | I84 |
| Hypotension | I95 |
| Acute bronchitis, unspecified | J20.9 |
| Bronchitis, not specified as acute or chronic | J40 |
| Emphysema, unspecified | J43.9 |
| Other chronic obstructive pulmonary disease | J44 |
| Asthma, unspecified | J45.9 |
| Pleural effusion, not elsewhere classified | J90 |
| Other spontaneous pneumothorax | J93.1 |
| Other disorders of lung | J98.4 |
| Gastro-oesophageal reflux disease with oesophagitis | K21.0 |
| Ulcer of oesophagus | K22.1 |
| Gastro-oesophageal laceration-haemorrhage syndrome | K22.6 |
| Chronic gastritis, unspecified | K29.5 |
| Other gastritis | K29.6 |
| Gastritis, unspecified | K29.7 |
| Angiodysplasia of stomach and duodenum with haemorrhage | K31.82 |
| Diaphragmatic hernia with obstruction, without gangrene | K44.0 |
| Noninfective gastroenteritis and colitis, unspecified | K52.9 |
| Angiodysplasia of colon without mention of haemorrhage | K55.21 |
| Vascular disorder of intestine, unspecified | K55.9 |
| **Paralytic ileus and intestinal obstruction without hernia** | K56 |
| **Diverticular disease of large intestine without perforation or abscess** | K57.3 |
| Constipation | K59.0 |
| Other specified diseases of anus and rectum | K62.8 |
| Polyp of colon | K63.5 |
| Cholelithiasis | K80 |
| Acute pancreatitis | K85 |
| Cellulitis | L03 |
| Generalized skin eruption due to drugs and medicaments | L27.0 |
| Decubitus ulcer | L89 |
| Ulcer of lower limb, not elsewhere classified | L97 |
| Pyogenic arthritis, unspecified | M00.9 |
| Gout, unspecified | M10.9 |
| Other primary coxarthrosis | M16.1 |
| **Coxarthrosis, unspecified** | M16.9 |
| **Primary arthrosis of other joints** | M190 |
| **Pain in joint** | M25.5 |
| **Torticollis** | M43.6 |
| **Unspecified spondylosis** | M47.9 |
| **Collapsed vertebra, not elsewhere classified** | M48.5 |
| **Cervicalgia** | M54.2 |
| **Low back pain** | M54.5 |
| **Other dorsalgia** | M54.8 |
| **Dorsalgia, unspecified** | M54.9 |
| **Other specified disorders of muscle** | M62.8 |
| **Olecranon bursitis** | M70.2 |
| **Synovial cyst of popliteal space [Baker]** | M71.2 |
| **Rotator cuff syndrome** | M75.1 |
| **Pain in limb** | M79.6 |
| **Unspecified osteoporosis with pathological fracture** | M80.9 |
| **Osteomyelitis** | M86 |
| Acute renal failure | N17 |
| Chronic kidney disease | N18 |
| Urethral stricture, unspecified | N35.9 |
| Hyperplasia of prostate | N40 |
| Inflammatory disorders of scrotum | N49.2 |
| Abnormal uterine and vaginal bleeding, unspecified | N93.9 |
| Pharyngeal pouch | Q38.7 |
| **Dyspnoea** | R06.0 |
| **Pain in throat and chest** | R07 |
| **Pleurisy** | R09.1 |
| **Abdominal and pelvic pain** | R10 |
| **Nausea and vomiting** | R11 |
| **Intra-abdominal and pelvic swelling, mass and lump** | R19.0 |
| **Paraesthesia of skin** | R20.2 |
| **Rash and other nonspecific skin eruption** | R21 |
| **Changes in skin texture** | R23.4 |
| **Cramp and spasm** | R25.2 |
| **Retention of urine** | R33 |
| **Other and unspecified symptoms and signs involving the urinary system** | R39.8 |
| **Somnolence** | R40.0 |
| **Disorientation, unspecified** | R41.0 |
| **Other and unspecified symptoms and signs involving cognitive functions and awareness** | R41.8 |
| Fever, unspecified | R50.9 |
| Malaise and fatigue | R53 |
| **Enlarged lymph nodes, unspecified** | R59.9 |
| **Localized oedema** | R60.0 |
| **Glycosuria** | R81 |
| **Abnormal findings on diagnostic imaging of lung** | R91 |
| Superficial injury of head | S00 |
| Open wound of head | S01 |
| Contusion of eyeball and orbital tissues | S05.1 |
| **Unspecified injury of head** | S09.9 |
| **Contusion of thorax** | S20.2 |
| **Fracture of rib(s), sternum and thoracic spine** | S22 |
| **Traumatic pneumothorax** | S27.0 |
| **Unspecified injury of thorax** | S29.9 |
| **Other superficial injuries of abdomen, lower back and pelvis** | S30.8 |
| **Open wound of lower back and pelvis** | S31.0 |
| **Fracture of lumbar spine and pelvis** | S32 |
| Sprain and strain of lumbar spine | S33.5 |
| Contusion and haematoma of kidney | S37.01 |
| **Other specified injuries of abdomen, lower back and pelvis** | S39.8 |
| **Fracture of upper end of humerus** | S42.2 |
| **Unspecified injury of shoulder and upper arm** | S49.9 |
| **Open wound of elbow** | S51.0 |
| **Fracture of forearm** | S5250 |
| **Contusion of other parts of wrist and hand** | S60.2 |
| **Open wound of wrist and hand part, part unspecified** | S61.9 |
| **Contusion of hip** | S70.0 |
| **Fracture of femur** | S72 |
| **Unspecified injury of hip and thigh** | S79.9 |
| Contusion of knee | S80.0 |
| Open wound of lower leg | S81 |
| **Tear of meniscus, current** | S83.2 |
| **Unspecified injury of ankle and foot** | S99.9 |
| **Injury, unspecified** | T14.9 |
| **Poisoning by antiparkinsonism drugs and other central muscle-tone depressants** | T42.8 |
| **Toxic effect of organic solvents: other organic solvents** | T52.8 |
| **Toxic effect of pesticides: herbicides and fungicides** | T60.3 |
| **Heat exhaustion, unspecified** | T67.5 |
| **Hypothermia** | T68 |
| **Angioneurotic oedema** | T78.3 |
| **Infection and inflammatory reaction due to other cardiac and vascular devices, implants and grafts** | T82.7 |
| **Other specified complications of cardiac and vascular prosthetic devices, implants and grafts** | T82.8 |
| **Infection and inflammatory reaction due to prosthetic device, implant and graft in urinary system** | T83.5 |
| **Supplementary code related to causes of morbidity & mortality classified elsewhere: Health service area** | Y92.22 |
| **Examination and observation following other accident** | Z04.3 |
| **Follow-up examination after surgery for malignant neoplasm** | Z08.0 |
| **Follow-up examination after other treatment for other conditions** | Z09.8 |
| **Attention to surgical dressings and sutures** | Z48.0 |
| **Other specified surgical follow-up care** | Z48.8 |
| **Care involving dialysis** | Z49.0 |
| **Chemotherapy session for neoplasm** | Z51.1 |
| Other specified medical care | Z51.8 |
